# Fatty Acid Potassium Had Beneficial Bactericidal Effects and Removed *Staphylococcus aureus* Biofilms while Exhibiting Reduced Cytotoxicity towards Mouse Fibroblasts and Human Keratinocytes

**DOI:** 10.3390/ijms20020312

**Published:** 2019-01-14

**Authors:** Takayoshi Kawahara, Miki Takita, Akihiro Masunaga, Hayato Morita, Tadayuki Tsukatani, Kohji Nakazawa, Daisuke Go, Sadanori Akita

**Affiliations:** 1 Shabondama Soap Co., Ltd., Kitakyushu 808-0195, Japan; kawahara@shabon.com (T.K.); miki-takita@shabon.com (M.T.); akihiro-masunaga@shabon.com (A.M.); h-morita@shabon.com (H.M.); 2 Department of Microbiology, University of Occupational and Environmental Health, Kitakyushu 807-0804, Japan; 3 Biotechnology and Food Research Institute, Fukuoka Industrial Technology Center, Kurume 839-0861, Japan; tukatani@fitc.pref.fukuoka.jp; 4 Department of Life and Environment Engineering, The University of Kitakyushu, Kitakyushu 808-0135, Japan; nakazawa@kitakyu-u.ac.jp (K.N.); y7mab007@eng.kitakyu-u.ac.jp (D.G.); 5 Department of Plastic Surgery, Wound Repair and Regeneration, Fukuoka University, Fukuoka 814-0180, Japan

**Keywords:** fatty acid potassium, biofilm, less cytotoxicity, bactericide, wound

## Abstract

Wounds frequently become infected or contaminated with bacteria. Potassium oleate (C18:1K), a type of fatty acid potassium, caused >4 log colony-forming unit (CFU)/mL reductions in the numbers of *Staphylococcus aureus* and *Escherichia coli* within 10 min and a >2 log CFU/mL reduction in the number of *Clostridium difficile* within 1 min. C18:1K (proportion removed: 90.3%) was significantly more effective at removing *Staphylococcus aureus* biofilms than the synthetic surfactant detergents sodium lauryl ether sulfate (SLES) (74.8%, *p* < 0.01) and sodium lauryl sulfate (SLS) (78.0%, *p* < 0.05). In the WST (water-soluble tetrazolium) assay, mouse fibroblasts (BALB/3T3 clone A31) in C18:1K (relative viability vs. control: 102.8%) demonstrated a significantly higher viability than those in SLES (30.1%) or SLS (18.1%, *p* < 0.05). In a lactate dehydrogenase (LDH) leakage assay, C18:1K (relative leakage vs. control: 108.9%) was found to be associated with a significantly lower LDH leakage from mouse fibroblasts than SLES or SLS (720.6% and 523.4%, respectively; *p* < 0.05). Potassium oleate demonstrated bactericidal effects against various species including *Staphylococcus aureus, Escherichia coli, Bacillus cereus*, and *Clostridium difficile*; removed significantly greater amounts of *Staphylococcus aureus* biofilm material than SLES and SLS; and maintained fibroblast viability; therefore, it might be useful for wound cleaning and peri-wound skin.

## 1. Introduction

Wounds increase healthcare costs, and a pivotal report has estimated that the US healthcare system spends an estimated 25 billion dollars annually treating chronic wounds alone [1]. Chronic wounds persist for an average of one year, frequently recur in up to 70% of subjects, and can lead to functional impairment and decreased quality of life. In addition, lower extremity wounds are a significant cause of morbidity [2], and diabetic foot ulcers are becoming more prevalent and more difficult to treat as well as being associated with high treatment costs [3]. Wounds frequently become infected or contaminated with bacteria. *Staphylococcus aureus* is known to form biofilms and to colonize the skin of caregivers as well as wounds, making it a leading cause of healthcare-associated infections, whereas *Clostridium difficile* forms spores that make it more resistant to conventional antimicrobial therapy.

In contrast, surgical site infections (SSIs) are a major complication of surgery and are related to longer hospital stays and higher hospital costs. Antiseptic solutions are widely used for preoperative bathing or showering in the belief that they help prevent SSIs from developing.

In eyelid preoperative cleansing, the use of baby shampoo was as good as povidone-iodine or isopropyl alcohol in a prospective, randomized, and comparative study [4] and skin cleansing with the topical use of chlorhexidine gluconate (CHG) was able to reduce the risk of central-line-associated bloodstream infection, SSIs, and the colonization of vancomycin-resistant entecocci (VRE) or methicillin-resistant *Staphylococcus aureus* (MRSA) in a systematic review [5]; however, the use, safety, and effectiveness of natural soap has not been reported in the literature.

In a previous study, the effects of 4% CHG, an antiseptic alone; CHG and bathing; or other hygiene products such as soap on SSIs were examined; however, CHG did not demonstrate superior efficacy [6]. In a surgical intensive care unit, daily skin cleansing with a bar of soap was found to exhibit comparable efficacy to CHG against bloodstream infections via central lines [7], which might be attributed to the fact that MRSA isolates display a reduced susceptibility to CHG [8].

A Markov model in chronic venous leg ulcers (VLUs) was constructed to evaluate the cost and clinical benefits (healing and decreased infection rates) of the two treatment modalities over a 1-year period using data from four randomized, controlled clinical studies (RCTs) included in a late Cochrane review and the cost data from a recently published economic evaluation of VLUs. Costs were calculated using 2014 United States dollars; wound outcomes included complete healing in 212 patients reported in the Cochrane meta-analysis and quality-adjusted life years (QALYs) with utility values obtained from 200 patients with VLUs calculated using standard gamble. Treatment with CI, Cadexomer Iodine, over one year was $7259 compared to $7901 for SC. This resulted in a cost saving of $643/patient in favor of CI when compared with SC, Standard Care. More patients treated with CI (61%) had their wounds healed when compared to the 54% treated with SC. Furthermore, patients treated with CI + SC experienced six additional ulcer-free weeks compared to patients treated with SC alone (i.e., 25 ulcer- free weeks compared to 19 ulcer-free weeks, respectively). Overall, CI resulted in 0.03 more QALYs (i.e., 0.86 QALYs compared to 0.83 for SC). The use of CI in addition to SC compared to SC alone over 52 weeks resulted in more wounds being healed and more QALYs along with a decrease of overall costs Therefore, the results of this study suggest that CI is cost effective when compared to SC alone in the management of patients with chronic VLUs [9].

Disinfection and the effective removal of pathogenic bacteria such as *Staphylococcus aureus*, its resistant form MRSA, and biofilm-forming MRSA, are important to prevent infections and provide wound care. We sought to investigate whether natural soap, which is devoid of any preservatives, additives, or synthetic materials, or its core ingredients such as fatty acid potassium could be used for such purposes. Thus, the cytotoxicity, bactericidal activity, and ability to remove MRSA of various types of fatty acid potassium were investigated.

## 2. Results

### 2.1. Structures of Fatty Acid Potassium and Synthetic Surfactant Detergents

**Figure 1:**
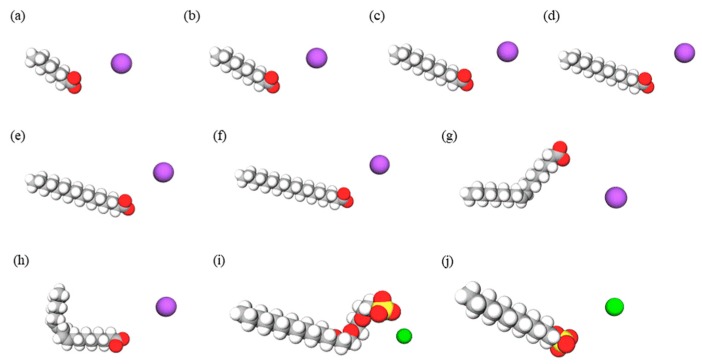
Structures of the fatty acid potassium molecules and synthetic surfactant detergents. (**a**) Potassium caprylate (C8K); (**b**) potassium caprate (C10K); (**c**) potassium laurate (C12K); (**d**) potassium myristate (C14K); (**e**) potassium palmitate (C16K); (**f**) potassium stearate (C18K); (**g**) potassium oleate (C18:1K); (**h**) potassium linoleate (C18:2K); (**i**) SLES; and (**j**) SLS. Carbon: gray, hydrogen: white, oxygen: red, sulfur: yellow, potassium ions: purple, sodium ions: green

Various different types of fatty acid potassium were examined in this study, and the synthetic surfactant detergents sodium lauryl ether sulfate (SLES) and sodium lauryl sulfate (SLS) were used as controls (Figure 1).

### 2.2. Cytotoxic Assays

To assess cytotoxicity, lactate dehydrogenase (LDH) leakage was measured and cell viability was determined using the WST (water-soluble tetrazolium) assay.

#### 2.2.1. LDH Leakage

**Figure 2:**
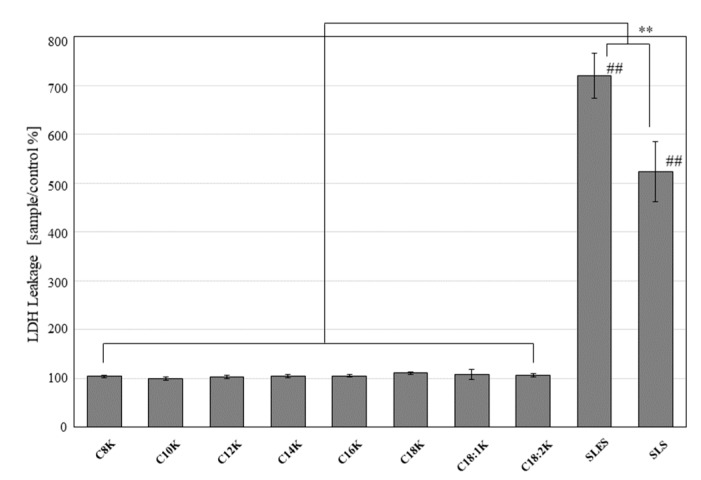
LDH leakage of BALB/3T3 cells treated with 0.5 mM fatty acid potassium, SLES, or SLS for 5 min. Untreated cells were used as the control. The percentage LDH leakage was calculated relative to the control value. Results are expressed as mean ± standard deviation (SD) values (*n* = 8). ** *p* < 0.01; ## *p* < 0.01 compared to the control.

After 5 min of treatment, the BALB/3T3 cells treated with the test solutions demonstrated significantly less LDH leakage than the SLES- or SLS-treated BALB/3T3 cells (percentage relative to the untreated control: potassium caprylate [C8K]: 104.1 ± 3.3%; potassium caprate [C10K]: 100.2 ± 3.3%; potassium laurate [C12K]: 103.9 ± 3.5%; potassium myristate [C14K]: 104.9 ± 3.6%; potassium palmitate [C16K]: 105.8 ± 3.2%; potassium stearate [C18K]: 111.0 ± 3.3%; potassium oleate [C18:1K]: 108.9 ± 10.2%; potassium linoleate [C18:2K]: 107.0 ± 3.2%; SLES: 720.6 ± 45.8%; and SLS: 523.4 ± 61.4%; *p* < 0.05 for all test substances vs. SLES and SLS) (Figure 2).

**Figure 3:**
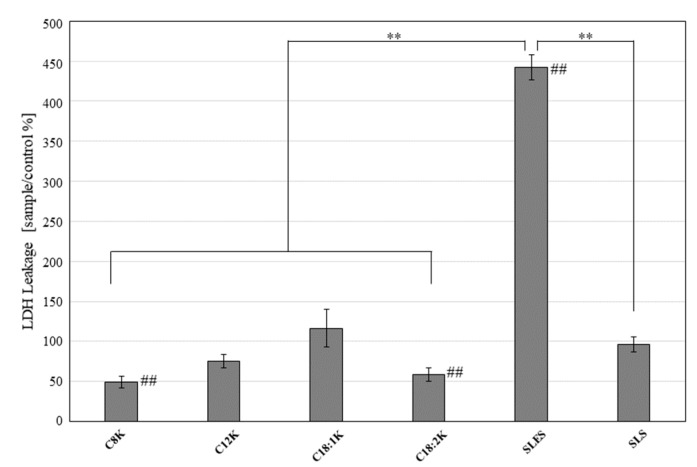
LDH leakage of NHEK-Ad cells treated with 0.5 mM fatty acid potassium, SLES, or SLS for 5 min. Untreated cells were used as the control. The percentage LDH leakage was calculated relative to the control value. Results are expressed as mean ± standard deviation (SD) values (*n* = 8). ** *p* < 0.01; ## *p* < 0.01 compared to the control.

After 5 min of treatment, NHEK-Ad cells treated with the test solutions demonstrated significantly less LDH leakage than the SLES-treated NHEK-Ad cells (percentage relative to the untreated control: potassium caprylate [C8K]: 49.2 ± 7.4%; potassium caprate [C10K]: 44.5 ± 9.9%; potassium laurate [C12K]: 75.3 ± 8.4%; potassium oleate [C18:1K]: 116.6 ± 23.4%; potassium linoleate [C18:2K]: 58.5 ± 8.6%; SLES: 442.3 ± 15.5%; and SLS: 96.5 ± 9.5%; *p* < 0.01 for all test substances vs. SLES) (Figure 3).

#### 2.2.2. Cell Viability

**Figure 4:**
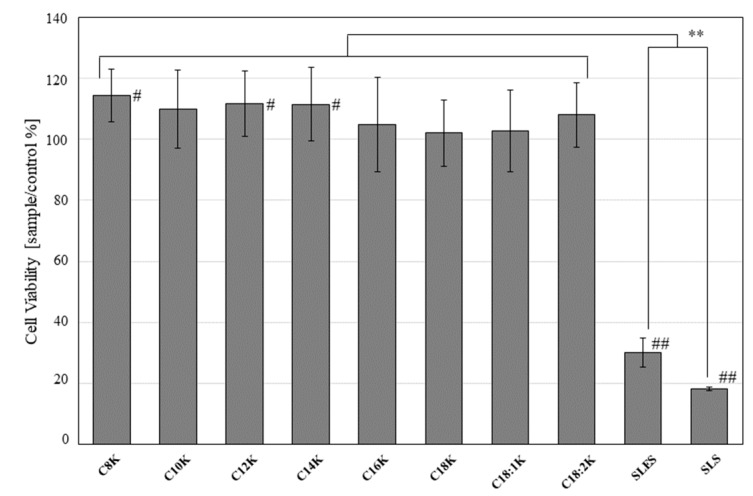
Viability of BALB/3T3 cells that were cultured in Dulbecco’s modified Eagle’s medium (DMEM) for 24 h after they had been treated with 0.5 mM fatty acid potassium, SLES, or SLS for 5 min. Untreated cells were used as the control. Percentage cell viability was calculated relative to the control value. Results are expressed as mean ± SD values (*n* = 8). ** *p* < 0.01, # *p* < 0.05 and ## *p* < 0.01 compared to the control.

BALB/3T3 cells were cultured for 24 h, then treated with one of the test substances for 5 min. The WST assay indicated that the cells treated with fatty acid potassium exhibited significantly greater viability than those treated with SLES or SLS (percentage viability relative to untreated control: untreated control: potassium caprylate [C8K]: 114.4 ± 8.7%; potassium caprate [C10K]: 109.9 ± 12.7%; potassium laurate [C12K]: 111.5 ± 10.7%; potassium myristate [C14K]: 111.5 ± 12.0%; potassium palmitate [C16K]: 104.8 ± 15.4%; potassium stearate [C18K]: 102.0 ± 10.7%; potassium oleate [C18:1K]: 102.8 ± 13.4%; potassium linoleate [C18:2K]: 108.0 ± 10.6%; SLES: 30.1 ± 4.8%; and SLS: 18.1 ± 0.6%; *p* < 0.05 for all test substances vs. SLES and SLS) (Figure 4).

**Figure 5:**
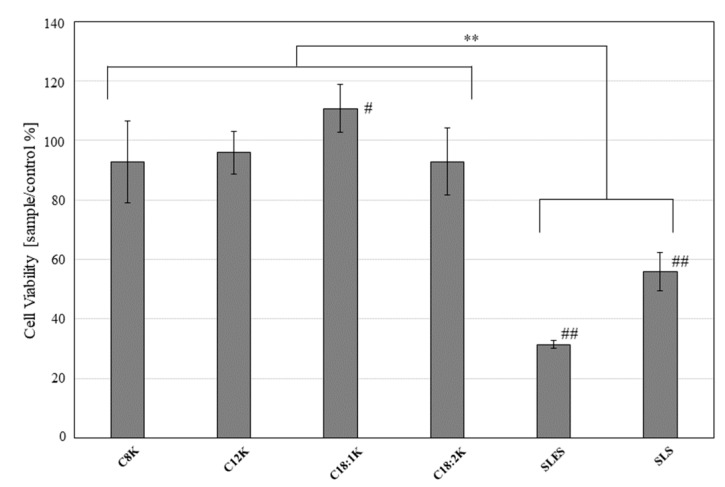
Viability of NHEK-Ad cells treated with 0.5 mM fatty acid potassium, SLES, or SLS for 5 min. Untreated cells were used as the control. Percentage cell viability was calculated relative to the control value. Results are expressed as mean ± SD values (*n* = 6). ** *p* < 0.01, # *p* < 0.05 and ## *p* < 0.01 compared to the control.

NHEK-Ad cells were cultured for 48 h, then treated with one of the test substances for 5 min. The WST assay indicated that the cells treated with fatty acid potassium exhibited significantly greater viability than those treated with SLES or SLS (percentage viability relative to untreated control: potassium caprylate [C8K]: 92.8 ± 13.7%; potassium caprate [C10K]: 60.9 ± 9.9%; potassium laurate [C12K]: 95.9 ± 7.1%; potassium oleate [C18:1K]: 110.7 ± 8.1%; potassium linoleate [C18:2K]: 92.9 ± 11.3%; SLES: 31.5 ± 1.3%; and SLS: 56.0 ± 6.4%; *p* < 0.05 for all test substances vs. SLES ) (Figure 5).

### 2.3. Anti-Bacterial Test

The bactericidal effects of fatty acid potassium (potassium oleate [C18:1K]) and an alcohol-based disinfectant on various bacterial suspensions were compared over 10 min. Regarding *Staphylococcus aureus*, potassium oleate exhibited significantly weaker bactericidal activity than the alcohol-based disinfectant at 1 min (reduction in bacterial number: 1.7 ± 0.9 vs. >4.5 ± 0.3 log colony-forming units (CFU)/mL, *p* < 0.05), but both treatments displayed similar levels of bactericidal activity from 5 to 10 min.

Both treatments caused consistent reductions (of ≤0.3 log CFU/mL) in the numbers of *Bacillus cereus* from 1 to 10 min.

Potassium oleate demonstrated consistently greater bactericidal activity against *Clostridium difficile* than the alcohol-based disinfectant from 1 to 10 min (bacterial number reductions: 1 min: >2.4 ± 0.5 vs. 0.4 ± 0.2, *p* < 0.05; 5 min: >2.4 ± 0.5 vs. 0.5 ± 0.3, *p* < 0.05; 10 min: >2.4 ± 0.5 vs. 0.5 ± 0.2, *p* < 0.01; potassium oleate vs. the alcohol-based disinfectant, respectively) (Table 1).

### 2.4. Effects on Biofilm-Forming MRSA

#### 2.4.1. Crystal Violet Assay

**Figure 6:**
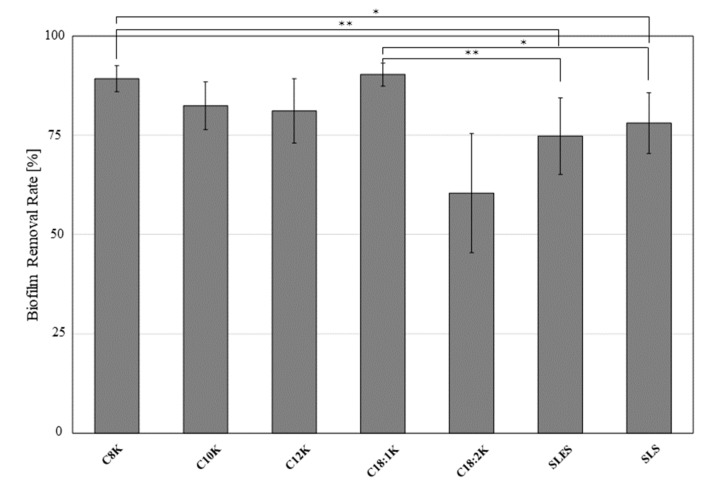
Percentage MRSA OJ-1 biofilm removal values of various types of fatty acid potassium, SLES, and SLS. The formed biofilm was placed in contact with the test substance for 1 min. The biofilm remaining in the well was stained with 0.1% crystal violet and quantified based on the absorbance at 570 nm using a microplate reader. The results are expressed as mean ± SD values (*n* = 6). * *p* < 0.05, ** *p* < 0.01.

By examining the amount of 0.1% crystal violet left after biofilms were placed in contact with one of five different fatty acids (potassium caprylate [C8K], potassium caprate [C10K], potassium laurate [C12K], potassium oleate [C18:1K], and potassium linoleate [C18:2K]), SLES, or SLS for 1 min, it was demonstrated that C18:1K (percentage biofilm clearance relative to control: 90.3 ± 2.9%) was significantly more effective against biofilm-forming MRSA than SLES (74.8 ± 9.7%, *p* < 0.01) and SLS (78.0 ± 7.7%, *p* < 0.05). In addition, potassium caprylate (C8K) demonstrated a significantly greater ability to remove the MRSA biofilm than SLES and SLS (89.3 ± 3.3%, *p* < 0.01 and 0.05, respectively) (Figure 6).

#### 2.4.2. Ultra-Microstructural Appearance after Treatment with Potassium Oleate (C18:1K)

**Figure 7:**
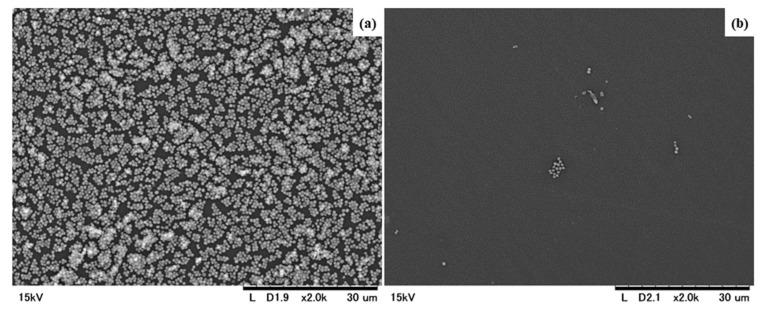
Scanning electron microscopy. Scanning electron microscopy micrographs of the untreated (**a**) and C18:1K-treated (**b**) biofilms that formed on the test pieces (2000×). MRSA biofilms formed on the test pieces after they had been incubated for 24 h at 37 °C. The MRSA biofilms that formed on the test pieces were removed by C18:1K. (**a**) An untreated biofilm that formed on a test piece; (**b**) A biofilm that had been treated with C18:1K for 1 min.

Scanning electron microscopy demonstrated that the MRSA biofilm that formed after 24 h incubation had almost completely disappeared after 1 min of treatment with potassium oleate (Figure 7).

## 3. Discussion

As the use of antiseptics or antibiotics can lead to secondary resistance, new methods are required to prevent and effectively treat wound infections. A biocompatible, non-ionic, completely water-soluble surfactant polymer dressing was found to improve wound healing via the direct effects on Gram-negative bacteria such as *Pseudomonas aeruginosa*. Specifically, it was shown to affect the lipid-based outer membranes of *Pseudomonas aeruginosa* and *Staphylococcus aureus* by disrupting their biofilm matrices and membrane glycoproteins, which decreased their metabolic activity [10]. Confocal laser-scanning microscopy confirmed that the same surfactant-based wound dressing caused the effective detachment and dispersion of bacterial biofilms including those produced by *Staphylococcus aureus* and MRSA in 48-h in vitro in the Center for Disease Control and Prevention biofilm reactor, filter biofilm, and chamber slide biofilm models, even in the absence of 1% silver sulfadiazine, an antimicrobial agent [11]. In our study, *Escherichia coli*, *Staphylococcus aureus*, and *Bacillus cereus* were incubated for 48 h before being treated with potassium oleate or the control (an alcohol-based detergent) for 10 min. Both of these treatments led to similar reductions in the numbers of these bacteria. The numbers of both *Escherichia coli* and *Staphylococcus aureus* were reduced by 4-fold, but *Bacillus cereus* was resistant to both substances. *Bacillus cereus* can produce protective endospores, and thus, is even resistant to novel antimicrobial agents such as platelet (PLT)-poor plasma, PLT-rich plasma, PLT gel, and solvent/detergent-treated PLT lysate biomaterials [12].

In the current study, potassium oleate caused a 2.4-fold reduction in the number of *Clostridium difficile* CFU within 1 min, whereas the alcohol-based disinfectant caused a 0.4-fold reduction in the number of *Clostridium difficile* CFU during this period. In children with severe burns, *Clostridium difficile*-associated diarrhea resulted in a 5-fold greater in-hospital mortality rate, longer hospital stays relative to the percentage total burnt body surface area, and prolonged acidosis due to diarrhea [13]. Thus, the use of potassium oleate rather than alcohol-based disinfectants is recommended in cases of *Clostridium difficile*-related infections.

In a study involving the in vitro decellularization of mouse skin, a detergent-free method was compared with non-ionic and anionic detergent methods, and sulfated glycosaminoglycan content was only significantly reduced (*p* < 0.05) in the ionic detergent treatment group. In contrast to the detergent-free method, all of the detergent-based methods caused significant reductions in mechanical scaffold strength and elastin content (*p* < 0.05) [14]. In the latter experiment, although fibroblasts were removed by both the detergent-free method and the non-ionic, synthetic, and anionic detergents, the detergents caused reductions in scaffold strength and the amount of extracellular matrix tissue, which is harmful and can adversely affect the condition of the remnant tissue. In our experiment, fatty acid potassium, which is found in anionic detergents, was demonstrated to be significantly less cytotoxic to mouse fibroblasts and human keratinocytes than SLES after treatment for 24 and 48 h. In this experiment, the viability of the mouse fibroblasts and human primary keratinocytes were assessed using the WST assay and LDH leakage assay, which were shown to be useful for assessing the cytotoxicity of substances towards human glioma stem cells (U87) and endothelial cells (HUVEC) in a microfluidic system [15]. In mouse fibroblasts, there were significantly less cytotoxic and more viability in all fatty acids than SLS, whereas in human primary keratinocytes, there was significantly greater cell viability among the selective fatty acids such as C8K, C12K, C18:1K, and C18:2K than SLS, but no LDH leakage. This may be due to the interaction between SLS and human keratinocytes (NCTC2455 keratinocyte cell line) in the photoallergy test [16].

In a case-control study of trauma in the US military, the occurrence of a polymicrobial infection was reported to be a significant risk factor for persistent infections. Therefore, biofilm production by multiple clinically important bacterial strains is significantly associated with persistent wound infections [17]. A type of MRSA (ATCC, BAA-2856; formerly named OJ-1) that we isolated from a clinical sample exhibited increased resistance to vancomycin in a mouse dermal chip model [18,19]. In addition, it was highly capable of producing highly virulent biofilms, and was able to survive in mouse Kupffer cells [20]. In the biofilm removal rate experiment conducted in the current study, which involved the above-mentioned biofilm-forming MRSA, C18:1K was significantly more effective at biofilm removal than SLES (by 15%, *p* < 0.01) and SLS (by 12%, *p* < 0.05). Another fatty acid potassium, C8K, was also significantly more effective at biofilm removal than SLES and SLS. Thus, other types of fatty acid potassium apart from potassium oleate are more effective at removing biofilms than SLES and SLS. In a previous study, SLS demonstrated activity against *Staphylococcus epidermidis*, *Staphylococcus aureus*, and *Pseudomonas aeruginosa*, as did oleic acid and benzalkonium chloride, in musculocutaneous wounds [21]. In contrast, SLES is an ingredient in cosmetics and has been shown to have cytotoxic effects [22], even though a shower gel containing SLES was demonstrated to transiently dampen inflammation and reduce bacterial growth in suction-blistered wounds [23]. Below the critical micelle concentration (0.1 mg/mL), SLS has minimal influence on the enzymatic activity of collagenase [24] and is also known to penetrate skin and cause cutaneous irritation. Cumulative SLS treatment significantly increased the concentration of this compound in the underlying epidermis, at least in vivo [25]. In contrast to these synthetic anionic detergents, the toxicity of fatty acid potassium and sodium oleate can change in systems that mimic natural rivers; i.e., it is higher upstream, but lower downstream, due to cation-dependent detoxification (forming metallic soaps) caused by the precipitation of sodium oleate in hard water. The toxic levels of oleate and palmitate salts were found to be 10-fold lower than those of laurate and myristate salts. When river water and local tap water were used for culturing, the toxic levels of all fatty acid salts were markedly higher (by 30- to 100-fold); i.e., the substances were less toxic than when ultra-pure water was used [26]. Therefore, potassium oleate, which is a type of fatty acid potassium, could be a useful wound-cleansing agent as it exhibits bactericidal activity, is effective at removing MRSA, is less toxic against host cells, and displays a beneficial eco-toxicity profile.

## 4. Materials and Methods

### 4.1. Reagents

Lauric acid, myristic acid, palmitic acid, stearic acid, oleic acid, and glutaraldehyde were purchased from Tokyo Chemical Industry Co. Ltd. (Tokyo, Japan). Caprylic acid, capric acid, linoleic acid, SLS, sodium chloride (NaCl), disodium hydrogen phosphate 12-water (Na_2_HPO_4_•12H_2_O), potassium dihydrogen phosphate (KH_2_PO_4_), sodium chloride (KCl), potassium hydroxide (KOH), hydrochloric acid (HCl), manganese (II) chloride tetrahydrate (MnCl_2_•4H_2_O), magnesium sulfate heptahydrate (MgSO_4_•7H_2_O), iron (II) sulfate heptahydrate (FeSO_4_•7H_2_O), calcium chloride dihydrate (CaCl_2_•2H_2_O), agar, crystal violet, Hank’s balanced salt solution (HBSS)(+) and 99.5% ethanol (EtOH) were purchased from FUJIFILM Wako Pure Chemical Corporation (Osaka, Japan). SLES was purchased from the NOF Corporation (Tokyo, Japan). The Dulbecco’s phosphate-buffered saline (d-PBS)(+) preparation reagent (with Ca and Mg) (100×) and fetal bovine serum (FBS) were purchased from Nacalai Tesque (Kyoto, Japan) and Thermo Fisher Scientific K.K. (Tokyo, Japan), respectively. WELPAS^®^ antiseptic solution for hands (0.2%), an alcohol-based disinfectant, was purchased from Maruishi Pharmaceutical Co. Ltd. (Osaka, Japan).

### 4.2. Preparation of Surfactant Solutions for the Cytotoxicity Assay

A total of eight different fatty acids, potassium caprylate (C8K), potassium caprate (C10K), potassium laurate (C12K), potassium myristate (C14K), potassium palmitate (C16K), potassium stearate (C18K), potassium oleate (C18:1K), and potassium linoleate (C18:2K), and the synthetic surfactants SLES and SLS were used in the cytotoxicity assays. d-PBS(+) or HBSS(+) buffer, containing Ca and Mg, was used to produce the fatty acid solutions. All fatty acid salts (C8K, C10K, C12K, C14K, C16K, C18K, C18:1K, and C18:2K; all 0.5 mM) were prepared by mixing the relevant fatty acid (C8, C10, C12, C14, C16, C18, C18:1, and C18:2, respectively) with KOH that had been solubilized with d-PBS(+) or HBSS(+) at 80 °C. Then, the pH of each fatty acid salt was adjusted to 10.4 by adding KOH. SLES and SLS (0.5 mM) were prepared by diluting them with d-PBS(+) or HBSS(+). Both of these solutions had final pH values of 7.7.

### 4.3. Preparation of Surfactant Solutions for the Antibacterial Test

C18:1K and an alcohol-based disinfectant were used in the antibacterial test. Oleic acid (315 mmol) and purified water were stirred while being heated. An equimolar amount of KOH was added and heated at 80 °C for one hour to convert the oleic acid to potassium oleate (C18:1K). Finally, the pH of the aqueous 315 mM C18:1K solution was adjusted to 10.4 by adding KOH. The alcohol-based disinfectant was used as is.

### 4.4. Preparation of Surfactant Solutions for the Biofilm Removal Test

A total of five different fatty acids, C8K, C10K, C12K, C18:1K, and C18:2K, and the synthetic surfactants SLES and SLS were used in the biofilm removal test. All fatty acid salts (C8K, C10K, C12K, C18:1K, and C18:2K; all 31.2 mM) were prepared by mixing the relevant fatty acid (C8, C10, C12, C18:1, and C18:2, respectively) with KOH that had been solubilized with purified water at 80 °C. Then, the pH of each fatty acid salt was adjusted to 10.4 by adding KOH. SLES and SLS (31.2 mM) were prepared by diluting them with purified water. The final pH values of the SLES and SLS solutions were 4.2 and 7.0, respectively.

### 4.5. Cell Culture

A permanent mouse fibroblast cell line, BALB/3T3 clone A31 cells (JCRB Cell Bank, Tokyo, Japan, JCRB9005). and a primary human keratinocyte cell line, NHEK-Ad cells (Lonza Japan Ltd., Tokyo, Japan) were cultured in DMEM (Thermo Fisher Scientific) supplemented with FBS (10%), 100 U/mL penicillin (Meiji Seika Pharma Co. Tokyo, Japan), and 100 μg/mL streptomycin (Meiji Seika Pharma) [27,28] and in KBM-Gold medium (Lonza Japan), respectively. In total, 1 × 10^4^ cells were added to each well of a 96-well Nunc MicroWell microplate (Thermo Fisher Scientific) before being cultured for 24 h for BALB/3T3 and 48 h for NHEK-Ad cells in a humidified atmosphere containing 5% CO_2_ at 37 °C [29].

### 4.6. Cytotoxicity Assay

**Figure 8:**
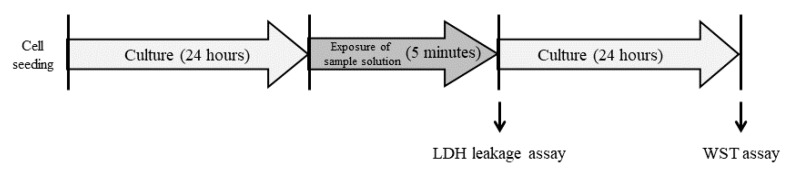
Cytotoxicity test procedure for the BALB/3T3 cells. BALB/3T3 cells that had been cultured in DMEM for 24 h were treated with 100 mL/well of each sample for 5 min. The solution was collected from each well and used for the LDH leakage assay. After the cells had been cultured for 24 h, their viability was assessed.

**Figure 9:**
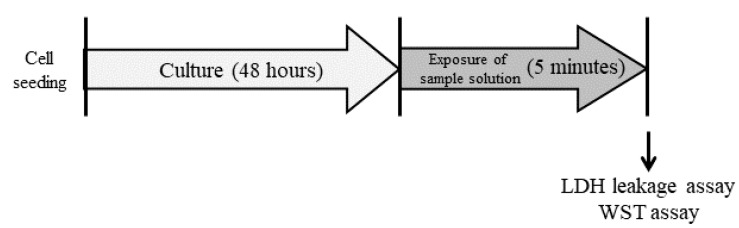
Cytotoxicity test for the NHEK-Ad cells. NHEK-Ad cells that had been cultured in KBM-Gold medium for 48 h were treated with 100 mL/well of each sample for 5 min. The solution was collected from each well and used for the LDH leakage assay. After that, their viability was assessed.

LDH leakage and cell viability were evaluated as indices of cytotoxicity [15]. BALB/3T3 cells were cultured for 24 h and treated with 100 μL/well of 0.5 mM C8K, C10K, C12K, C14K, C16K, 18K, 18:1K, C18:2K, SLES, or SLS for 5 min at room temperature. d-PBS(+)-treated cells were used as the control. Then, the solution was collected from each well and used for the LDH leakage assay. BALB/3T3 cells were washed five times with the culture medium and then cultured for 24 h. After being cultured, BALB/3T3 cells were washed five times with the culture medium, and then their viabilities were evaluated (Figure 8). NHEK-Ad cells were cultured for 48 h and treated with 100 μL/well of 0.5 mM C8K, C10K, C12K, 18:1K, C18:2K, SLES, or SLS for 5 min at room temperature. HBSS(+)-treated cells were used as the control and either the LDH assay or washed 3-times following the viability assay (Figure 9). LDH leakages and cell viabilities were assessed in each well using the cytotoxicity LDH assay kit-WST (Dojindo Laboratories, Kumamoto, Japan) and cell counting kit-8 (Dojindo Laboratories), respectively.

### 4.7. Preparation of the Bacterial Suspensions

*Escherichia coli* NBRC3972, *Staphylococcus aureus* subsp. *aureus* NBRC12732, and *Bacillus cereus* NBRC15305 were purchased from the National Institute of Technology and Evaluation (Tokyo, Japan), and *Clostridium difficile* ATCC9689 was purchased from Kanto Chemical Co. Inc. (Tokyo, Japan).

*Escherichia coli* and *Staphylococcus aureus* subsp. *aureus* were cultured on soybean casein digest agar (SCDA; Nissui Seiyaku Co., Tokyo, Japan) at 30–35 °C for 18–24 h. Several colonies were then added to 10 mL of Trypticase soy broth (TSB; bioMerieux Japan Ltd., Tokyo, Japan) and incubated with shaking at 35 °C for 18–24 h. Each bacterial suspension was diluted to 10^7^–10^8^ CFU/mL by adding purified water.

The culture media for *Bacillus cereus* and *Clostridium difficile* were prepared by adding 784 mL of ion exchanged water to 32 g of Trypticase soy agar (TSA; bioMerieux Japan Ltd.), which was sterilized by autoclaving and then maintained at around 50 °C. Next, an 8 mL solution of 0.306 g MnCl_2_•4H_2_O, 2.5 g MgSO_4_•7H_2_O, and 0.003 g FeSO_4_•7H_2_O dissolved in 100 mL of 0.01 mol/L HCl and an 8-mL solution of 1.5 g CaCl_2_•2H_2_O dissolved in 100 mL of 0.01 mol/L HCl were added and mixed. *Bacillus cereus* and *Clostridium difficile* were inoculated into the culture medium and cultured at 30–35 °C until sufficient spore formation could be confirmed. After the culturing procedure, sterile purified water was added, and a suspension was created with a sterile bacterial spreader. The suspension liquid was collected and heat-treated for 15 min at 75 °C, before being immediately cooled and then diluted with purified water to about 10^7^–10^8^ CFU/mL.

### 4.8. Anti-Bacterial Test

The anti-bacterial test was performed by thoroughly mixing 0.2 mL of the bacterial suspension with 20 mL of C18:1K or the alcohol-based disinfectant at room temperature [30]. Samples were taken from the reaction mixture after 1, 5, and 10 min and were neutralized by diluting them 100 times with soya casein digest lecithin polysorbate (SCDLP) broth (Eiken Chemical Co. Ltd., Tokyo, Japan). Ten-fold dilutions of neutralized samples of *Escherichia coli, Staphylococcus aureus*, and *Bacillus cereus* were cultured on SCDA at 30–35 °C for 40–48 h. Ten-fold dilutions of neutralized samples of *Clostridium difficile* were cultured on Columbia agar plates at 30–35 °C for 48–72 h. The number of colonies was counted and expressed as CFU/mL. Bactericidal activity was expressed as the log CFU reduction in the viable count compared with the log CFU viable count seen after the addition of purified water.

### 4.9. Biofilm Formation

The MRSA (OJ-1, ATCC No. BAA-2856™) was provided by Shiro Jimi (Central Laboratory for Pathology and Morphology, Faculty of Medicine, Fukuoka University, Fukuoka, Japan) and was cultured on a TSA slant for 24 h at 37 °C. Several colonies were then diluted with TSB to an optical density at 600 nm of 0.1, and 1 mL of the prepared suspension was then added to each well of a 12-well cell culture plate (Corning Inc., Corning, NY, USA). After the plates had been cultured for 24 h at 37 °C to encourage biofilm formation [31], the resultant biofilms were washed with saline to remove any planktonic cells.

### 4.10. Biofilm Removal Test

To measure the biofilm removal rate, 1 mL of the relevant sample was brought into contact with the biofilm for 1 min at room temperature [32]. To remove any planktonic cells, the biofilm was washed three times with saline. Subsequently, the biofilm remaining in the well was stained with 0.1% crystal violet for one hour [33] and then washed with saline twice. After being decolorized with ethanol, the amount of biofilm remaining was quantified based on the absorbance at 570 nm, which was evaluated with a Synergy H4 hybrid microplate reader (BioTek Instruments Inc., Winooski, VT, USA). The biofilm removal rate was determined using the following equation:

$$\mathrm{Biofilm}\;\mathrm{removal}\;\mathrm{rate}\;\left(\%\right)=\frac{\left(A_{c}-A_{b}\right)-\left(A_{s}-A_{b}\right)}{\left(A_{c}-A_{b}\right)}\times 100$$

where *A_c_*, *A_b_*, and *A_s_* are the absorbance of the control, blank, and sample, respectively

### 4.11. Biofilm Structure According to Scanning Electron Microscopy (SEM)

The test pieces used measured 8 mm × 8 mm and were obtained from a Petri dish for cells (Corning Inc.). The sterilized test pieces were placed in each well of a 12-well cell culture plate. The prepared bacterial suspension (1.5 mL) was added to each well and then incubated for 24 h at 37 °C to allow biofilms to form on the test pieces. Subsequently, the biofilm removal test was performed using these test pieces according to the above-mentioned procedure. The biofilm was rinsed with saline to remove any loosely adherent cells, and then fixed in 2.0% glutaraldehyde solution for 2 h at 4 °C. Subsequently, the biofilms were dehydrated through a graded series of ethanol solutions to 99.5%, before being air-dried overnight and coated with platinum/palladium ions for the SEM analysis. The SEM was carried out with a Miniscope TM-1000 electron microscope (Hitachi, Tokyo, Japan).

### 4.12. Statistical Analysis

Data were presented as mean ± SD values. Statistical analyses were performed using repeated-measures analysis of variance for the LDH leakage and WST assays, and the Student’s *t*-test for the antibacterial and biofilm removal tests. *p*-Values of <0.05 were considered statistically significant in each analysis.

## 5. Conclusions

Potassium oleate, one of the types of fatty acid potassium found in natural soap, can kill various bacteria including MRSA, and can also effectively remove biofilm-forming MRSA. As for its effects on normal host cells, it caused significantly less LDH leakage and significantly lower reductions in cell viability when compared with SLES and SLS, which are synthetic detergents.

Thus, potassium oleate might contribute to the provision of cost-effective wound care.

## Figures and Tables

**Table 1:** Bactericidal activity of C18:1K and an alcohol-based disinfectant.

	C18:1K (log CFU/mL)			Alcohol-Based Disinfectant (log CFU/mL)		
	1 min	5 min	10 min	1 min	5 min	10 min
*Escherichia coli*	3.3 (0.4)	>4.3 (0.2)	>4.3 (0.2)	>4.3 (0.2)	>4.3 (0.2)	>4.3 (0.2)
*Staphylococcus aureus*	1.7 (0.9) *	>4.4 (0.1)	>4.4 (0.2)	>4.5 (0.3)	>4.4 (0.3)	>4.4 (0.2)
*Bacillus cereus*	0.2 (0.2)	0.2 (0.3)	0.3 (0.3)	0.1 (0.2)	0.2 (0.3)	0.2 (0.4)
*Clostridium difficile*	>2.4 (0.5) *	>2.4 (0.5) *	>2.4 (0.5) **	0.4 (0.2)	0.5 (0.3)	0.5 (0.2)

Anti-bacterial testing was performed by mixing 0.2 mL of each bacterial suspension with 20 mL of the relevant test substance. Results are expressed as mean ± SD values (*n* = 3). * *p* < 0.05, ** *p* < 0.01.

## Abbreviations

MRSA	Methicillin-resistant *Staphylococcus aureus*
SLES	Sodium lauryl ether sulfate
SLS	Sodium lauryl sulfate
SSI	Surgical site infection
